# Molecular Characterization of Hepatitis Delta Virus in the Western Amazon, Acre, Brazil

**DOI:** 10.3390/v18070730

**Published:** 2026-06-30

**Authors:** Rutilene Barbosa Souza, Thor Oliveira Dantas, Luiz Fellype Alves de Souza, Tárcio P. Roca, Adrhyan Araújo, Jackson A. S. Queiroz, Ana M. Passos-Silva, Mariana Araújo Costa, Edna Maria Gomes Gonçalves, Luis Edgardo Riveros Aguilar, Ana Alice Maia Gonçalves, Alexsandro Sobreira Galdino, Daniel Archimedes da Matta, Deusilene Vieira, Carlos Brites

**Affiliations:** 1 Programa de Pós-Graduação em Medicina e Saúde, Universidade Federal da Bahia, Salvador 40110-040, Brazil; crbrites@gmail.com; 2 Laboratório Rodolphe Mérieux, Centro de Infectologia Charles Mérieux, Rio Branco 69917-672, Brazil; fellype.alves@cicm-brasil.org (L.F.A.d.S.); luisriveros433@gmail.com (L.E.R.A.); darchimedes@hotmail.com (D.A.d.M.); 3 Fundação Hospitalar Governador Flaviano Melo, Rio Branco 69917-672, Brazil; thor.mdac@gmail.com (T.O.D.); ednaacre2004@gmail.com (E.M.G.G.); 4 Centro de Ciências da Saúde e Desporto, Universidade Federal do Acre, Rio Branco 69920-900, Brazil; 5 Laboratório de Virologia Molecular, Fundação Oswaldo Cruz Rondônia—FIOCRUZ/RO, Porto Velho 76812-245, Brazil; tarcioroca@aluno.fiocruz.br (T.P.R.); adrhyan.oliveira@fiocruz.br (A.A.); jackson.queiroz@fiocruz.br (J.A.S.Q.); ana.maisa@fiocruz.br (A.M.P.-S.); deusilene.vieira@fiocruz.br (D.V.); 6 Programa de Pós-Graduação em Biologia Experimental, Universidade Federal de Rondônia e Fundação Oswaldo Cruz Rondônia—UNIR/FIOCRUZ/RO, Porto Velho 76801-974, Brazil; 7 Laboratório Central de Saúde Publica do Acre, Rio Branco 69918-504, Brazil; mariana_araujoc@hotmail.com; 8 Laboratório de Biotecnologia de Microrganismos, Universidade Federal de São João Del Rei, Campus Centro Oeste, Divinópolis 36307-352, Brazil; anafish@hotmail.com (A.A.M.G.); asgaldino@ufsj.edu.br (A.S.G.)

**Keywords:** HDV-3, hepatitis delta, phylogeography, viral diversity, Western Amazon

## Abstract

Background: Hepatitis delta (HDV) is the most severe form of chronic viral hepatitis, with rapid progression to cirrhosis and hepatocellular carcinoma, and remains neglected in the Amazon Basin. The state of Acre, in the Western Brazilian Amazon, is endemic for HDV, with a high prevalence of genotype 3 (HDV-3). This study aimed to analyze epidemiological data, quantify HDV RNA, identify genotypes, and describe the phylogeny and phylogeography of HDV in Acre. Methods: This cross-sectional study included patients positive for HBsAg and anti-HDV under follow-up at the Specialized Assistance Service (SAE) in Rio Branco, Acre, between March and November 2023. Blood samples were collected for HDV RNA quantification using one-step RT-qPCR. Samples with detectable viremia (Ct ≤ 30) underwent Nested-PCR, Sanger sequencing, phylogenetic analysis (Maximum Likelihood), temporal signal evaluation, and Bayesian phylogeographic reconstruction. Results: Among 108 patients (median age 43; 55.6% female), HDV RNA was detected in 48.1%, with viral loads ranging from 140 to 24,000,000 copies/mL. Of these, 55.8% had >100,000 copies/mL. Genotyping (*n* = 41) identified exclusively HDV-3. Phylogenetic analysis revealed genetic heterogeneity among HDV-3 isolates, with the formation of two major phylogenetic clades. Bayesian analysis estimated tMRCA around 1818 and suggested dispersion from Amazonas to Acre and neighboring regions. Conclusion: HDV-3 predominates in Acre with high genetic diversity, indicating sustained viral circulation in the Western Amazon and reinforcing the need for improved surveillance and diagnosis.

## 1. Introduction

Hepatitis delta, also known as hepatitis D, is an infectious disease that affects the liver and is the most severe type of chronic hepatitis, with rapid progression to hepatic cirrhosis and hepatocarcinoma [1]. The hepatitis delta virus (HDV) [2] belongs to the family Kolmioviridae and is the only representative of the genus Deltavirus [3]. HDV is classified as a satellite virus of hepatitis B virus (HBV) due to its dependence on the Hepatitis B Virus Antigens (HBsAg), present in the viral envelope of HBV, for a productive infection in humans [4,5,6].

HDV was described for the first time by Rizzetto et al. in 1977, being identified as a delta antigen while analyzing liver biopsies from patients infected with HBV [7]. Structurally, HDV consists of a circular single-stranded RNA genome (~1.7 kb) that contains self-cleaving ribozyme domains and is associated with hepatitis delta antigen (HDAg) [3]. Currently, eight HDV genotypes (HDV-1 to HDV-8) have been identified [8,9]. These genotypes differ by approximately 35–40% at the nucleotide level and exhibit characteristic geographic distributions across different regions of the world [10]. Some regions show high endemicity of HDV, such as in Central Africa, the Middle East, and parts of Asia, Eastern Europe, the Mediterranean and the Americas [11,12,13]. In South America specifically, the Amazon Basin has the highest prevalence of HDV-3 in the world. This genotype is considered the most aggressive genotype among all [14]. As expected, the number of HDV cases is closely related to the high prevalence of HBV in populations [15,16]. In Brazil, over the last 23 years, the North region accounted for 72.5% of the 4525 cases of hepatitis delta reported to the Ministry of Health. Regarding hepatitis B, the capital cities of Rondônia and Acre States presented the highest rates of HBV infection throughout the country [16].

The Amazon Basin, encompassing Brazil, Peru, Colombia, Venezuela, and Ecuador, remains one of the world’s most important endemic regions for HDV infection [17,18,19]. In Brazil, most reported cases occur in the Northern region, although universal HBV vaccination programs introduced since the 1990s have substantially reduced HDV incidence among younger populations [20,21,22]. Currently, HDV screening in Brazil is performed through anti-HDV testing among HBsAg-positive individuals, with molecular confirmation by HDV-RNA detection, although important logistical challenges remain in remote and cross-border regions of the Amazon Basin [16,23].

In South America, outbreaks of severe liver disease in indigenous and riverside populations, such as “Sierra Nevada Hepatitis” and “Santa Marta Black Fever” in Colombia [24,25] and “Black Lip Fever” and “Lábrea Hepatitis” in the Brazilian Amazon [26], were later associated with HDV infection and have been described since the 1980s, with high mortality rates and fast-evolving microepidemics being reported [27,28]. The Amazon Basin harbors the highest prevalence of HDV-3 worldwide [15], and nowadays, it is known that there is an association between HDV genotype 3 and fulminant hepatitis outbreaks in the Amazon Basin [29].

Hepatitis delta is a public health problem that affects local populations, remaining a neglected disease. There is a lack of knowledge about the circulation of HDV in this region and its transmission dynamics over time, despite previous epidemiological and molecular studies addressing HDV circulation in the Amazon region and South America [15,30,31]. Molecular and phylogenetic studies are necessary to help understand these issues, in addition to the geolocation of isolates from HDV infections in the state of Acre, considering that many of these patients who seek care in cities with better healthcare facilities come from rural and remote forest areas.

The present study aims to analyze epidemiological data from patients with HDV, characterize the molecular profile through the HDV RNA quantification test and identification of genotypes, determine the most prevalent genotypes and describe the phylogeny and phylogeography based on the HDV sequences.

## 2. Materials and Methods

### 2.1. Study Design

This is a cross-sectional study with samples collected from patients being monitored for chronic hepatitis B and D (delta) at the Specialized Assistance Service (SAE) in the city of Rio Branco, state of Acre.

### 2.2. Patients and Samples

The study included all patients with positive serology for HBsAg and anti-HDV who are being monitored for hepatitis B and D at SAE of the Fundação Hospitalar Governador Flaviano Melo (FUNDHACRE). These chronic patients were routinely followed at the healthcare service for hepatitis B and hepatitis delta care and were consecutively invited to participate in the present study between March and November 2023. During this period, clinical and epidemiological data were collected, and blood samples were collected in separator gel tubes for serum extraction and subsequent HDV molecular analyses, including quantitative HDV RNA testing, which was not routinely available outside the study setting. Only samples collected during the study period were included, and no previously stored samples were used. Patients were previously screened by total anti-HDV serology and subsequently underwent HDV-RNA detection and quantification using the previously validated RT-qPCR assay described by Queiroz et al., 2023 [32]. All patients included in the study followed the ethical procedures determined by the research ethics committee (CAAE 59822421.4.0000.5009). The samples were sent to the CICM/LRM (Charles Mérieux Center/Rodolphe Mérieux Laboratory) at FUNDHACRE, then aliquoted and frozen at −80 °C until the moment of laboratory tests. Samples showing detectable viremia (CT ≤ 30) were sent to the Molecular Virology Laboratory at FIOCRUZ/RO for genotyping.

### 2.3. Exclusion Criteria

HBsAg-positive patients who did not present reactive anti-HDV serology results, regardless of the therapy adopted or stage of disease progression, or who did not have complete clinical/epidemiological data, were excluded from this analysis.

### 2.4. Statistical Analysis

Statistical analyses were performed using IBM SPSS Statistics version 32 (IBM Corp., Armonk, NY, USA). Categorical variables were compared between HDV-RNA-positive and -negative patients using Pearson’s chi-square test or the Fisher–Freeman–Halton exact test, as appropriate. A *p*-value < 0.05 was considered statistically significant.

### 2.5. Viral RNA Extraction

Viral RNA was extracted using magnetic beads, using an automated extractor (EXTRACTA 32 DNA and RNA extractor, LOCCUS, São Paulo, Brazil). For extraction and purification performance, the EXTRACTA KIT FAST—Viral DNA/RNA extraction kit (MVXA-P016FAST) was used following the manufacturer’s instructions, with a final eluted product of 50 µL.

### 2.6. Quantification of HDV RNA

Quantification of HDV-RNA was performed using the one-step multiplex RT-qPCR assay previously described by Queiroz et al., 2023 [32], in which primers and probes were designed to detect a conserved ribozyme region shared among the eight known HDV genotypes. Only samples with HDV-RNA Ct values ≤ 30 were selected for sequencing to ensure adequate sequence quality.

### 2.7. Reverse Transcription

Samples positive for HDV-RNA were subjected to reverse transcription for complementary DNA (cDNA) synthesis using SuperScript™ III Reverse Transcriptase enzyme (Thermo Fisher Scientific^®^, Waltham, MA, USA) associated with 0.5 μg of random primer, according to the manufacturer’s instructions.

### 2.8. Polymerase Chain Reaction (PCR)

To obtain complete HDV genome coverage, a modified amplification strategy previously described in the literature [33] was used. This approach uses primers to amplify the viral genome as only two overlapping fragments. Fragment 1 (F1; ~966 bp) was amplified using primers 320s (5′-CCAGAGGACCCCTTCAGCGAAC-3′) and 1267as (5′-GAAGGAAGGCCCTGGAGAACAAGA-3′), whereas Fragment 2 (F2; ~1216 bp) was amplified with primers 900s (5′-CATGCCGACCCGAAGAGGAAAG-3′) and 503as (5′-CCCCGGGATAAGCCTCACTCG-3′). Amplification was performed using Platinum™ SuperFi™ PCR Master Mix (Invitrogen, Waltham, MA, USA) under the following conditions: an initial denaturation at 98 °C for 30 s, followed by 40 cycles of 98 °C for 15 s, annealing at 60 °C (F1) or 58 °C (F2) for 30 s, extension at 72 °C for 45 s, and a final extension step at 72 °C for 5 min. Following amplification, PCR products were purified using the ExoSAP-IT PCR Product Cleanup Kit (Applied Biosystems™, Foster City, CA, USA).

### 2.9. Molecular Sequencing

The BigDye™ Terminator v1.1 Cycle Sequencing Kit (Applied Biosystems™, Foster City, California, USA) was used according to the manufacturer’s instructions for sequencing. The reaction product was purified using BigDye XTerminator™ Purification Kit (Applied Biosystems™, Foster City, CA, USA). The sequencing run was performed by the Rede de Plataformas Tecnológicas FIOCRUZ RPT09F -FIOCRUZ/RO using an automated Sanger sequencer, Seqstudio (Applied Biosystems, Waltham, MA, USA). The electropherogram was edited and analyzed using MEGA11-Molecular Evolutionary Genetic Analysis v.11.0 software [34]. After sequence assembly and quality control, near-complete HDV genomes ranging from approximately 1500 bp to 1678 bp were recovered and used for subsequent phylogenetic and evolutionary analyses.

### 2.10. Phylogenetic Analysis

The sequences generated in the present study were pairwise in the whole-genome alignments (*n* = 50) of HDV-3 collected through the public Genbank database available at the National Center for Biotechnology Information—NCBI [https://www.ncbi.nlm.nih.gov/]. Multiple sequence alignment was performed using online software MAFFT v.7 [35]. The general time-reversible (GTR) model of nucleotide substitution with a gamma-distributed rate variation among sites, four rate categories (G4), a proportion of invariable sites (I) and empirical base frequencies (F) were measured using the tool ModelFinder incluída no IQ-TREE v.2.2.2.6 [36]. Phylogenetic analysis was performed using the Maximum Likelihood (ML) method using the IQ-TREE v.2.2.2.6 software, and branch support values were obtained by bootstrapping 1000 replicates. The tree was edited and viewed by the program Figtree v1.4.4 (https://github.com/rambaut/figtree/releases/tag/v1.4.4, accessed on 24 March 2024).

### 2.11. Temporal Signal Estimation

To verify the existence of a temporal signal in the study data set, an analysis was carried out using the TempEst v.1.5.3 software using a “non-clock” phylogenetic tree obtained using IQtree v2.2.2.6. R software v.4.3.2 (https://www.r-project.org/, accessed on 24 March 2024) was used to plot the scatter plot.

### 2.12. Bayesian Analysis

A temporal tree was performed in the BEAUti software v.1.10.1. by including the date of collection for Bayesian inference. The uncorrelated relaxed clock was set, allowing each branch of the phylogenetic tree to have a different evolutionary rate than the others. The model GTR+F+G+I was defined as more suitable by the ModelFinder tool. The Bayesian SkyGrid coalescent model was used and thus defined 50 parameters of Tree Prior, with an end point of 100 years before the most recent sampling. The length of the Monte Carlo Markov Chain (MCMC) was defined as 3 108, with data collection every 30,000 data, which thus creates 10,000 samples, which was sufficient for parameter convergence. For running the race, BEAST v.1.10.1 was used.

Bayesian reconstruction and tMRCA determination were analyzed using the software Tracer v.1.7.1. The validation of the analysis was performed by a value of Effective Sample Size (ESS) > 200 for the parameters. The phylogenetic tree was summarized, and a consensus tree was created using TreeAnotator v.1.10.1., which in turn selects a single “target” tree and records it with the summary information of the entire sample of trees (10,000), excluding 10% of the samples as burn-in, creating a Maximum Clade Credibility (MCC) tree. The consensus tree was visualized and customized using FigTree v.1.4.3.

### 2.13. Phylogeographic Reconstruction in Continuous Space

A phylogeographic analysis was carried out in continuous space to analyze the dispersion of HDV from a cartographic point of view. The methodology used is similar to that used in the reconstruction of the Bayesian analysis with information on geographic coordinates of latitude and longitude of each city where the study individuals reside through the BEAUti v.1.10.4 software. Therefore, the Cauchy Relaxed Random Walk (RRW) model was selected for phylogeographic diffusion in continuous space, with an MCMC chain length of 3 × 108 and data collection every 30,000 data. Analysis results were summarized using TreeAnotator v.1.8.4. to build an MCC tree. This, in turn, was used to plot the diffusion results on the map using SPREAD v.1.0.7 software, and the result was viewed and edited using Google Earth Pro 7.3.6.9750 software.

## 3. Results

### 3.1. Epidemiological Characteristics of Participants

The study enrolled 108 patients with chronic hepatitis delta receiving care at FUNDHACRE, a referral center for viral hepatitis in the Western Brazilian Amazon, between March and November 2023. Among the participants, 70 (64.8%) were born in Acre State, 36 (33.3%) in Amazonas State, and two (1.9%) in other Brazilian states (Bahia and Ceará), reflecting the regional patient flow to the study center. The epidemiological data and molecular characteristics of HDV isolated from these patients were analyzed.

The median age was 43 years (IQR: 21–71 years), with a predominance of female patients (55.6%). Most participants were married or living with a partner (67.6%), and 87.9% were literate. A significant association was observed between age group and HDV-RNA detection, with younger individuals (<43 years) more frequently presenting detectable HDV-RNA (61.5% vs. 35.7%, *p* = 0.007) (Table 1).

**Figure 1:**
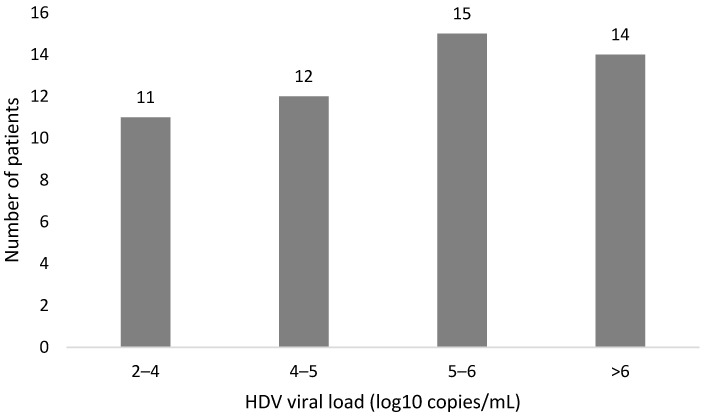
Distribution of HDV viral load among patients with detectable HDV-RNA according to viral load ranges expressed as log10 copies/mL.

HDV-RNA was detected in 52/108 (48.1%) patients. Among positive individuals, HDV viral load ranged from 1.44 × 10^2^ to 1 × 10^9^ copies/mL, with heterogeneous distribution across viral load categories (Figure 1).

HDV genomic sequences were successfully obtained from 42 of the 52 HDV-RNA-positive samples. All 42 HDV samples (100%) sequenced were classified as HDV-3.

### 3.2. Phylogeny and Phylogeography

Two geographic location criteria were evaluated: city of birth and city of residence. The majority of patients with a detectable viral load were born in the state of Acre (*n* = 34 or 65.4%); the others were born in the state of Amazonas (*n* = 18 or 34.6%). Currently, 52% (*n* = 27) live in cities other than those where they were born (Table 1), while the remaining 48% (*n* = 25) remain in the cities of their birth. Approximately 84.6% (*n* = 44) of patients live in the State of Acre, with the majority in the capital, Rio Branco. Meanwhile, some other patients reside in the state of Amazonas, in municipalities that border Acre, such as Boca do Acre, Pauini, Envira and Ipixuna.

**Figure 2:**
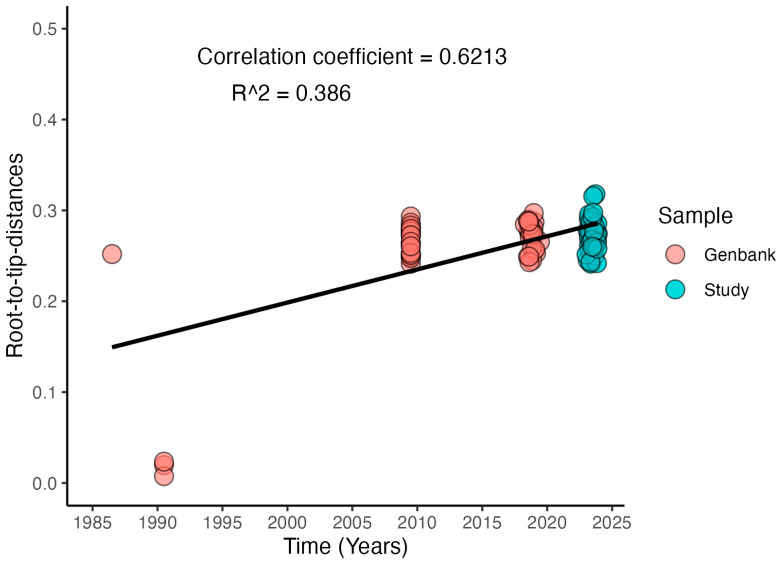
Root-to-tip regression analysis was used to evaluate the temporal signal of the HDV-3 dataset. The genetic distance from the phylogenetic root to each sequence is plotted against the corresponding sampling year. Sequences generated in the present study (*n* = 42) are represented by blue dots, whereas reference sequences retrieved from GenBank (*n* = 50) are represented by red dots. The regression analysis showed a positive correlation between genetic divergence and sampling time (R^2^ = 0.386; correlation coefficient = 0.6213), supporting the presence of sufficient temporal signal for molecular clock-based phylogenetic inference.

To assess the temporal signal of the dataset, a root-to-tip regression analysis was performed using a dataset composed of 92 HDV-3 sequences, including 42 sequences generated in the present study and 50 reference sequences retrieved from GenBank. A root-to-tip regression analysis was performed by correlating the genetic distance from the phylogenetic root to each sequence with its respective sampling date. A positive correlation was observed (R^2^ = 0.386; correlation coefficient = 0.6213), supporting the suitability of the dataset for subsequent molecular clock analyses (Figure 2).

**Figure 3:**
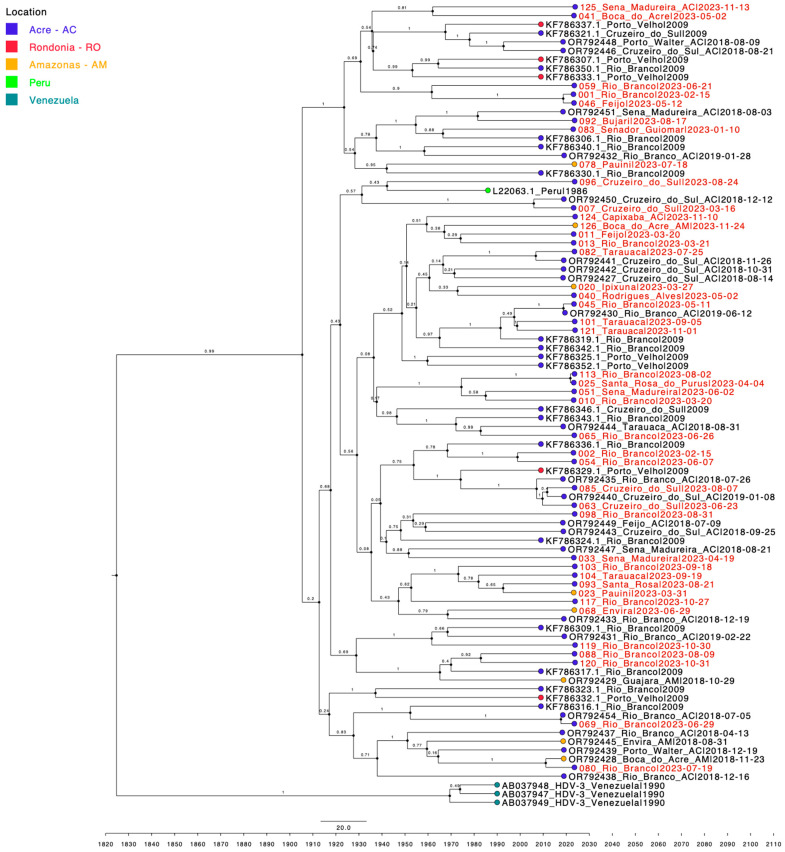
Bayesian maximum clade credibility (MCC) phylogenetic tree containing 42 study samples (highlighted in red) and the whole HDV genome sequencing retrieved from GenBank. The posterior probability values are contained in the branches. The time scale in years is shown at the bottom.

We evaluated the Bayesian Skygrid coalescent model to estimate the time of the Most Recent Common Ancestor (tMRCA), which was dated to the year 1818 (95% high posterior density—HPD 1714-1905). The Bayesian maximum clade credibility (MCC) phylogenetic tree (Figure 3) shows the study sequences, with 100% (42/42) classified as HDV-3. It was possible to observe two main clusters: the first containing samples from Rio Branco—AC together with other sequences from the state of Amazonas; and another cluster with sequences from Rio Branco, other cities in Acre and samples from nearby cities belonging to the states of Amazonas and Rondônia.

**Figure 4:**
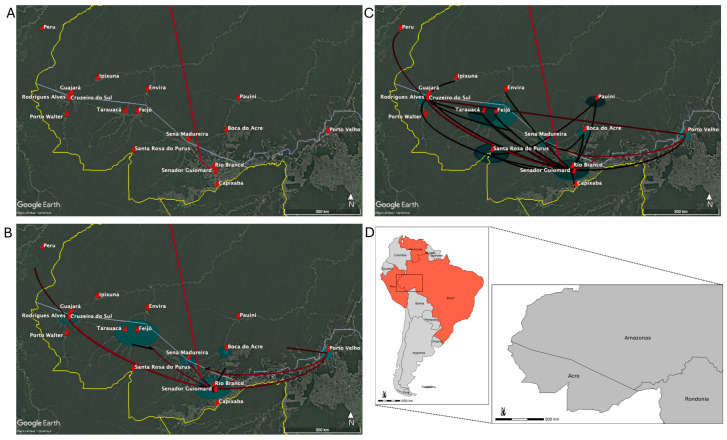
Continuous-space phylogeographic reconstruction of HDV-3 spread in South America based on 92 sequences. Panel (**A**) shows the inferred spatial diffusion of HDV-3 in 1920, representing the earliest dissemination events in the region. Panel (**B**) depicts the estimated viral spread in 1970, indicating a progressive expansion across the northern Brazilian states. Panel (**C**) illustrates the reconstructed phylogeographic pattern in 2023, highlighting the main diffusion routes among the Brazilian states of Acre (AC), Amazonas (AM), and Rondônia (RO), as well as connections with neighboring countries, including Venezuela and Peru (partially obscured). Panel (**D**) shows a reference map indicating the geographic location of the study area within the Western Amazon, including the Brazilian states AC, AM and RO, as well as neighboring countries relevant to the phylogeographic reconstruction. The shaded area highlights the state of Acre within the Western Amazon region.

The continuous-space phylogeographic reconstruction suggests that the ancestral lineage represented in our dataset may have originated in the Amazonas region during the early nineteenth century (tMRCA estimate), followed by dissemination toward Acre and neighboring areas of the Western Amazon (Figure 4A). However, given the temporal depth of the inference relative to the sampling period and the geographic composition of the dataset, these results should be interpreted cautiously and viewed as a reconstruction of the evolutionary history of HDV-3 within the Western Amazon rather than the entirety of Brazil.

By the 1920s, the virus reached the state of Acre, likely associated with human migration from southern Amazonas municipalities bordering Acre, such as Boca do Acre and Pauini, which are geographically close to Rio Branco (Figure 4A). During the 1940s, HDV expanded from Rio Branco to Cruzeiro do Sul (AC) and Porto Velho, the capital of Rondônia, indicating regional dissemination within the Western Amazon (Figure 4B). In the mid-1960s, viral spread extended to additional municipalities in Acre and to Peru, likely driven by cross-border movements originating in Acre (Figure 4B). From the 1980s onward, a broader dissemination pattern was observed, with HDV spreading across multiple localities throughout the state of Acre, consistent with the spatial diffusion pattern observed in the most recent reconstruction (Figure 4C).

## 4. Discussion

In the Amazon rainforest region, chronic viral hepatitis is a public health problem due to the lack of access to laboratory diagnostics and medical care [21]. The state of Acre is in this region, next to the borders with two other Brazilian states and the borders with Peru and Bolivia. The study area is classified as endemic for hepatitis delta due to the high prevalence of HDV in the Amazon region of Brazil [11,30,37] and neighboring countries such as Peru [23,38], Colombia [31] and Venezuela [39]. It is difficult to determine the real prevalence of HDV in the region, due to crucial factors such as locations that are difficult to access (some cities are accessible by air or river only).

The limitation to estimate the prevalence of HDV is discussed by Chen et al. (2021) [40], where it highlights that in low- and middle-income countries, health care is insufficient, which makes it difficult for early diagnosis and appropriate treatment in a timely manner. In a study conducted by Lago et al. (2018) [41], to evaluate the prevalence of HDV among HBsAg-positive patients in Brazil, the authors found the highest rates in the states of Acre and Amazonas, 24.3% and 13.8%, respectively. Regarding hepatitis B, in 2019, Melo da Silva et al. [42] conducted a study in patients with chronic HBV in the state capital of Acre, where they found a prevalence of 23.9% of HDV.

Additionally, younger individuals (<43 years) were more frequently represented among patients with detectable HDV-RNA (61.5% vs. 35.7%, *p* = 0.007). This finding may suggest ongoing viral circulation among younger age groups in the Western Amazon, although further studies are needed to better understand the factors associated with HDV replication and persistence.

The Ministry of Health recommends that HBsAg-positive patients should get the anti-HDV test, especially those from endemic areas of the Amazon region [43]. In other parts of the world, they follow the same recommendation for HDV diagnostic testing; however, in some cases, it is recommended to test individuals at risk, such as immigrants, injecting drug users and people who are vulnerable to sexually transmitted diseases [44,45].

In the present work, 108 patients with chronic delta hepatitis were enrolled in this study over an 8-month period in 2023, mostly born or living in rural areas. The majority of these patients are young, with a median age of 43 years. The female gender was prevalent (55.6%). Our findings in the general population of the study showed that 48.1% (52/108) of patients had detectable viral load. In Acre state, Melo da Silva et al., (2019) [42] in a correlation study between the genotypes of HBV and HDV viruses in patients treated for hepatitis, found 57.4% of RNA-HDV (54/94) with serology reagent for anti-HDV, while Silva et al., 2021 [46], in a cross-sectional study for the characterization of HBV and HDV viruses in illicit drug users in northern Brazil, detected 60% of RNA-HDV (9/15).

Among the eight genotypes of HDV described in Brazil, the genotype HDV-3 is predominant [41,46]. In this current study, 100% of samples were classified as HDV-3. Indeed, the HDV-3 genotype is the most prevalent in the Amazon region, ranging from 81.8% to 100% [15,42,46]. Although other genotypes have been identified in the region, HDV-1, HDV-5 and HDV-8 [11,37,46], we only identified the genotype HDV-3 in this study.

The HDV-3 sequences displayed intragenotypic genetic heterogeneity, characterized by their distribution across multiple branches and the formation of two major phylogenetic clusters. HDV is a small RNA virus showing greater genetic variability [47] and has a high mutation rate, estimated at 10^−3^ to 10^−4^ substitutions/site/year, which may contribute to high intragenotypic genetic variability [48]. Although all isolates belonged to HDV genotype 3, the observed clustering pattern suggests that the introduction of HDV in the state of Acre did not occur in just one single event. However, the available data do not allow us to distinguish conclusively between independent introductions and local diversification following a shared ancestral introduction. Therefore, both scenarios should be considered plausible explanations for the phylogenetic structure observed in the present study.

According to the phylogeographic reconstruction, the ancestral HDV-3 lineage represented in our dataset may have circulated in the Amazonas region since the nineteenth century, subsequently spreading to Acre and neighboring areas of the Western Amazon. Nevertheless, the analysis is more appropriately interpreted as a reconstruction of HDV-3 dissemination within the Western Amazon rather than evidence of the historical introduction of HDV into Brazil as a whole.

Around the 1920s, the HDV arrived in Acre probably due to migration of individuals from the municipalities of Amazonas that are on the border with the state of Acre, such as Boca do Acre and Pauini, which are close to the capital, Rio Branco. In the 1940s, from Rio Branco, HDV entered Cruzeiro do Sul (Acre) and Porto Velho, capital of the state of Rondônia. In the mid-1960s, HDV was inserted in other cities of Acre and in Peru, the latter probably from individuals from the state of Acre. Since the 1980s, HDV has spread to other regions of Acre. It was noted that some patients born in the Amazonas migrated to Acre State over time, especially to Rio Branco city. The study on the endemicity of HBV/HDV coinfection in an Amazon city in the Jurua Valley suggests that mobility between states around Acre may be culturally connected to relocation by waterway, specifically through the rivers Solimões, Acre, Purus, and Juruá [15].

Our findings are in agreement with those reported by Roca et al. (2024) [49], who demonstrated the long-term circulation and complex spatio-temporal dynamics of HDV-3 in the Western Brazilian Amazon [49]. The presence of distinct phylogenetic clusters observed in our dataset also supports the continued circulation of multiple HDV-3 lineages within the region. Importantly, our study expands upon these previous findings by incorporating newly generated near-complete genomes from Acre collected between 2021 and 2023, providing updated evidence of the ongoing endemic transmission and evolutionary diversification of HDV-3 in the Western Amazon.

The main limitation of the study is the lack of clinical data, outcomes and mortality rates of the patients enrolled in this study. The next stage of the study consists of evaluating the clinical correlation and progression for cirrhosis in patients. Overall, in this first phase of the study, we aimed to present the laboratory and demographic results, in addition to the quantification of RNA-HDV and intra-specific genotypic characterization of HDV in the Western Amazon region. Nonetheless, the results of an 8-month study that included over 108 patients receiving follow-up and treatment in the Brazilian public health system appear to be unprecedented in this part of northern Brazil, primarily due to the assessment of viral loads and the display of HDV genotypic characteristics in the state of Acre. Deeper historical inferences regarding the origin and introduction of HDV-3 should be interpreted cautiously, and broader conclusions regarding the dissemination of HDV throughout Brazil cannot be established from the present dataset alone.

## 5. Conclusions

The study presents a demographic profile of the participating patients and the molecular characterization of the HDV virus in the state of Acre and surrounding areas, with a focus on the distribution of genotypes and viral loads in the region. The results obtained throughout the study period demonstrate a high HDV viral load, highlighting the importance of quantitative molecular diagnosis. The study enables a more detailed understanding of HDV circulation in the area and provides data that support the development of public health strategies for effective patient management and treatment.

## Figures and Tables

**Table 1:** Demographic data from 108 patients with chronic hepatitis D enrolled in the study.

Variable	Patients *n* = 108	HDV-RNA Positive *n* = 52	HDV-RNA Negative *n* = 56	*p*-Value **
	n (%)	n (%)		
Age, years *				0.007
Age groups				
<43	52 (48.1)	32 (61.5)	20 (35.7)	
≥43	56 (51.9)	20 (38.5)	36 (64.3)	
**Gender**				0.263
Female	60 (55.6)	26 (50.0)	34 (60.7)	
Male	48 (44.4)	26 (50.0)	22 (39.3)	
**Marital status**				0.016
Married	73 (67.6)	42 (80.8)	31 (55.4)	
Single	23 (21.3)	7 (13.5)	16 (28.6)	
Divorced/Separated	5 (4.7)	0	5 (8.9)	
Widower	3 (2.8)	1 (1.9)	2 (3.6)	
Not informed	4 (3.7)	2 (3.8)	2 (3.6)	
**Education**				0.944
Illiterate	12 (11.1)	6 (11.5)	6 (10.7)	
Elementary Education	49 (45.4)	25 (48.1)	24 (42.9)	
High School	27 (25.0)	13 (25.0)	14 (25.0)	
Higher Education	11 (10.2)	4 (7.7)	7 (12.5)	
Not informed	9 (8.3)	4 (7.7)	5 (8.9)	

* Age is presented as median (IQR). ** Data are presented as n (%). Categorical variables were compared using Pearson’s chi-square test or Fisher–Freeman–Halton exact test, as appropriate. Statistical analyses were performed using IBM SPSS Statistics version 32. *p*-values < 0.05 were considered statistically significant.

## Data Availability

Sequences submitted in this study are available at NCBI GenBank with the accession numbers OR792427-OR792454.

## Abbreviations

The following abbreviations are used in this manuscript:

HDV	Hepatitis Delta Virus
SAE	Specialized Assistance Service
CICM	Centro de Infectologia Charles Mérieux
LACEN	Central Public Health Laboratory
tMRCA	Time to the Most Recent Common Ancestor
Ct	Cycle threshold
